# DRUG-INDUCED SLEEP ENDOSCOPY FOR TARGETED SLEEP SURGERY IN PEDIATRIC PATIENTS

**DOI:** 10.51894/001c.122918

**Published:** 2024-08-31

**Authors:** Ani Mnatsakanian

**Affiliations:** 1 Ear, Nose, and Throat (ENT) Macomb Oakland

## 47

### INTRODUCTION

Obstructive sleep apnea (OSA) and sleep-disordered breathing (SDB) are prevalent in 2-5% and up to 35% of the general pediatric population, respectively. Untreated OSA can lead to detrimental effects in children, including daytime sleepiness, poor cognition, cardiovascular derangements, neurobehavioral issues, and even growth abnormalities. Pediatric patients with OSA and SDB are commonly managed by surgical intervention targeting anatomic obstruction. Current recommendations suggest the removal of tonsils and adenoids as the first-line treatment of moderate-severe pediatric OSA. The current guidelines suggest drug induced sleep endoscopy (DISE) as a secondary measure should OSA persist after adenotonsillectomy. However, drug-induced sleep endoscopy has become an increasingly popular primary technique to evaluate the airway and the level of obstruction in children with clinically non-enlarged tonsils and adenoids.

### OBJECTIVES

This study aims to evaluate the efficacy of drug-induced sleep endoscopy (DISE) targeted surgery to identify the locations of obstruction and to determine how DISE findings influence whether the standard of care surgery, adenotonsillectomy, is performed.

### METHODS

This prospective cohort study was done at an academic children’s hospital. All patients (n = 42) underwent polysomnography. DISE was performed to evaluate tonsil and adenoid size, Yellon tongue base, lateral pharyngeal wall (LPW) collapse, and signs of laryngomalacia. Surgery was performed based on the most prominent locations of obstruction. Pre-operative and post-operative University of Michigan Pediatric Sleep Questionnaire (UMPSQ) was given to determine the likelihood of residual OSA.

### RESULTS

Surgeries included tonsillectomy, adenoidectomy, lingual tonsillectomy, laryngeal cleft repair, supraglottoplasty, and turbinate reduction. Patients had improvement in UMPSQ score from 13.36 ± 3.67 to 5.68 ± 3.46 (P=0.05). Those who underwent adenotonsillectomy had a greater decrease in UMPSQ scores than those who did not (P=0.03). Patients with significant LPW collapse were more likely to have adenotonsillectomy (P=0.001), while patients with higher Yellon tongue base scores were less likely (P=0.005). There was no statistically significant relationship between OSA severity and whether adenotonsillectomy was performed.

### CONCLUSIONS

DISE is a valuable tool for evaluating children with multi-level obstruction and findings change surgical decision-making for children without enlarged tonsils.

